# Relationship of Mycotoxins Accumulation and Bioactive Components Variation in Ginger after Fungal Inoculation

**DOI:** 10.3389/fphar.2017.00331

**Published:** 2017-06-02

**Authors:** Zhixin Yang, Haiwei Wang, Guangyao Ying, Meihua Yang, Yujiao Nian, Jiajia Liu, Weijun Kong

**Affiliations:** ^1^College of Pharmacy, Heilongjiang University of Chinese MedicineHarbin, China; ^2^Institute of Medicinal Plant Development, Chinese Academy of Medical Sciences and Peking Union Medical CollegeBeijing, China; ^3^College of Pharmacy, Jinzhou Medical UniversityJinzhou, China; ^4^College of Traditional Chinese Medicine, Jilin Agricultural UniversityChangchun, China

**Keywords:** ginger, fungal contamination, mycotoxin accumulation, component variation, UFLC-MS/MS, UPLC-PDA

## Abstract

Ginger has got increasing worldwide interests due to its extensive biological activities, along with high medical and edible values. But fungal contamination and mycotoxin residues have brought challenges to its quality and safety. In the present study, the relationship of content of mycotoxins accumulation and bioactive components variation in ginger after infection by toxigenic fungi were investigated for the first time to elucidate the influence of fungal contamination on the inherent quality of ginger. After being infected by *Aspergillus flavus* and *Aspergillus carbonarius* for different periods, the produced mycotoxins was determined by an immunoaffinity column clean-up based ultra-fast liquid chromatography coupled with tandem mass spectrometry, and the main bioactive components in ginger were analyzed by ultra performance liquid chromatography-photodiode array detection. The results showed that consecutive incubation of ginger with *A. flavus* and *A. carbonarius* within 20 days resulted in the production and accumulation of aflatoxins (especially AFB_1_) and ochratoxin A, as well as the constant content reduction of four bioactive components, which were confirmed through the scanning electron microscope images. Significantly negative correlation was expressed between the mycotoxins accumulation and bioactive components variation in ginger, which might influence the quality and safety of it. Furthermore, a new compound was detected after inoculation for 6 days, which was found in our study for the first time.

## Introduction

Ginger (*Zingiber officinale* Rosc.), a monocotyledon belonging to Family Zingiberaceae, is widely cultivated in tropical and subtropical countries and regions, such as China, India, Nigeria. As an important edible and medicinal matrix, it is not only considered as a dietary adjunct contributing to the taste and flavor of food, but also as a widely used traditional Chinese medicine (TCM) with anti-oxidant, anti-angiocarpy, anti-inflammation, and anti-microbial activities because of its abundant bioactive components like 6-gingerol, 8-gingerol, 10-gingerol, and 6-shogaol, etc. (Dugasani et al., 2010; Daily et al., 2015). But, previous studies have reported that ginger can be easily contaminated by fungi during the growth, processing, and storage progresses due to its internal factors and some unsuitable extrinsic environmental conditions to produce mycotoxins (Zinedine et al., 2006; Cao et al., 2013; Wen et al., 2014; Lippolis et al., 2017).

Mycotoxins are the secondary metabolites produced by toxigenic fungi, which can be found in a large number of matrices including foods, grains, feeds, spices, and TCMs, etc. They exhibit strong carcinogenicity, mutagenicity, and immunosuppression properties to pose serious threats, such as liver cancer, kidney failure, and effects on the nervous system to human health. Among these mycotoxins, aflatoxins (AFs), which are mainly produced by strains of *Aspergillus flavus* and *Aspergillus parasiticus*, are of great interest due to their high toxicity and incidence in many matrices including ginger. And aflatoxin B_1_ (AFB_1_) is the most commonly found and predominant one, which usually occupy about 90% of the AFs residue in contaminated foodstuffs (Jayashree and Subramanyam, 2000; Food and Agriculture Organization, 2004; Joint FAO/WHO Expert Committee on Food Additives, 2008). Due to its strongly hepatotoxic and hepatocarcinogenic properties, AFB_1_ has been classified as Group 1A carcinogen by the International Agency for Research on Cancer [IARC] (1999). In addition, ochratoxin A (OTA) is another easily detected mycotoxin in food, spices, and TCMs (Han et al., 2010; Wen et al., 2014; Zhao et al., 2014; Benites et al., 2017), which is a chronic nephrotoxin and do harm to the kidney function and has a sufficient evidence of carcinogenicity in experimental animal research and inadequate evidence in humans, as well as has been classified as Group 2B carcinogen by IARC (International Agency for Research on Cancer [IARC], 1999).

From then on, a lot of studies on the fungal contamination and mycotoxins production in ginger have been published (Trucksess et al., 2009; Iha and Trucksess, 2010; Wen et al., 2014; Ali et al., 2015; Yang et al., 2016; Lippolis et al., 2017). But, they mainly focused on the methods development, as well as their application in the detection of mycotoxins in ginger and related products. While, the influence of fungal contamination and mycotoxins residue in these ginger matrices was seldom involved. Our previous works have illustrated that fungal contamination and mycotoxins residue will lead to the content reduction of the active components of *Glycyrrhiza uralensis*, which might influence the inherent quality and safety of this TCM (Zhou et al., 2013, 2014). Therefore, it is in great need and necessity to explore the relationship of mycotoxins accumulation and bioactive components variation in ginger after being polluted by toxigenic fungi, further to exhibit the influences of fungal contamination and mycotoxins production on the inherent quality and safety of ginger and related products.

## Materials and Methods

### Materials

Dry gingers were purchased from Anguo Medicine Market (Hebei, China) and were identified by Professor Bengang Zhang (Institute of Medical Plant Development, Chinese Academy of Medical Science and Peking Union Medical College, Beijing, China). The samples were pulverized to pass through a 50-mesh sieve to get the ginger powder to be sealed and stored in plastic bags at -20°C before analysis.

One ochratoxigenic *Aspergillus carbonarius* (ITEM-5222) lyophilized powder was supplied by Agri-Food Toxigenic Fungi Culture Collection (ITEM) of the Institute of Science of Food Production, National Research Council (CNR) (Bari, Italy). Another aflatoxigenic *A. flavus* lyophilized powder (CGMCC 3.4410) was purchased from the China General Microbiological Culture Collection Center (Beijing, China). The two types of fungi were dissolved in 0.5 mL of sterile water for culture on Czapek Dox Agar (CDA) medium at constant temperature and humidity (28°C, 90% relative humidity, RH) for 7 days.

### Reagents and Chemicals

AFs standard including 2 μg AFB_1_, 2 μg AFG_1_, 0.5 μg AFB_2_, and 0.5 μg AFG_2_ in 1 mL of methanol was purchased from Qingdao Pribolab Biotechnology Co., Ltd. (Qingdao, China), and a standard solution of OTA (1 mg in 1 mL of methanol) was obtained from ALEXIS (Lausen, Switzerland) and gingerols standard (6-gingerol, 8-gingerol, 10-gingerol, and 6-shogaol) in the form of solid or oiliness were supplied by Chroma-Biotechnology Co., Ltd. (Chengdu, China).

Acetonitrile and methanol were both in chromatographic grade provided from Thermo Fisher Scientific (Beijing, China). Other reagents and chemicals were all analytical grades and purchased from Beijing Chemical Works (Beijing, China).

Standard solution stocks of 6-gingerol, 8-gingerol, 10-gingerol, and 6-shogaol were prepared respectively by dissolving accurately weighted content of standards in methanol and the mixed standard solution was in a concentration of 38.4 μg/mL for 6-gingerol, 40.8 μg/mL for 8-gingerol, 55.4 μg/mL for 10-gingerol, and 40.9 μg/mL for 6-shogaol.

Phosphate-buffered saline (PBS) was prepared by dissolving 0.2 g KCl, 0.2 g KH_2_PO_4_, 8 g NaCl, and 1.2 g Na_2_HPO_4_ in 1000 mL of water (pH was adjusted to 7.0 with 0.1 M HCl). Two percent Tween-20–PBS solution (PBST) was prepared by dissolving 0.2 g KCl, 0.2 g KH_2_PO_4_, 8 g NaCl, 1.2 g Na_2_HPO_4_, and 20 mL of Tween-20 in 1000 mL of water. The CDA medium, which was prepared by dissolving 2 g NaNO_3_, 1 g K_2_HPO_4_, 0.5 g MgSO_4_, 0.5 g KCl, 0.01 g FeSO_4_, 30 g sucrose, 15 g agar in 1000 mL of distilled water (pH 6.8 ± 0.2), was got from Aobox Biotechnology Co., Ltd. (Beijing, China).

### Fungal Spore Suspension Preparation

After incubation and growth in an incubator for 1 week, the fungal spores were suspended on 25 mL of CDA medium with the addition of 10 mL of sterile 0.01% Tween-20 solution. Then, the mycelium and spores mixture solution was removed by gravity filtration through at least three layers of autoclaved cheese cloth (Christensen et al., 2012). The concentration of spore suspension was determined and adjusted to 10^7^ cfu/mL with a hemocytometer slide (0.1 mm depth, 1/400 mm^2^) and an XDS-1B optical microscope (Chongqing, China) to get the standard spore suspension for subsequent use (Li et al., 2016).

### Sample Inoculation

Twenty grams of the sterilized ginger powder (under a UV lamp for 9 h) was introduced on a culture dish, followed by the addition of 1 mL of *A. flavus* and *A. carbonarius* homogeneous standard spore suspension and 10 mL sterile water on the center part of the dish. Thereafter, the dishes were sealed by parafilm and then were incubated in the incubator for 2, 4, 6, 8, 10, 12, 14, 16, 18, and 20 days at 28°C, 90% RH. Another 20 g of the sterilized ginger powder as a control was not infected with any fungi and cultured for 20 days under the same condition.

### Determination of Five Mycotoxins

#### Sample Preparation

The infected ginger powder and non-infected ginger powder collected at a 2-day interval were prepared for the analysis of four AFs and OTA. Five grams of inoculated or non-infected ginger powder and 1 g NaCl were weighed and placed in a 50-mL centrifuge tube, and then 25 mL of methanol/water (80:20, v/v) solution were added and the mixtures were extracted by ultrasonication in an ultrasonic cleaning bath at 500 W for 20 min. The extraction was centrifuged at 10,000 rpm for 5 min. Subsequently, 5 mL of the supernatant was transferred into another 50-mL centrifuge tube and diluted and mixed with 40 mL of 2% PBST solution. The mixed diluent was filtered through the 0.45 μm syringe filter and 40 mL of the filtrate was passed through an immunoaffinity column-aflatoxins (IAC-AFT)/OTA immunoaffinity column (Huaan Magnech Bio-Tech Co., Ltd., Beijing, China) and the target mycotoxins were absorbed on the column. The column was washed with 20 mL of PBS solution (pH 7.0) until 2–3 mL of air passed through it and the investigated toxins were finally eluted with 1.5 mL of methanol and collected in a 2-mL volumetric flask to the final volume. The solution was vortexed for 30 s and filtered through a 0.22-μm syringe filter and 2 μL of the filtrate was injected into the ultra fast liquid chromatography with tandem mass spectrometry (UFLC-MS/MS) system for analysis.

#### UFLC-MS/MS Conditions

An UFLC system (Shimadzu, Kyoto, Japan) coupled with a 5500 QTRAP^®^ hybrid triple quadrupole/near ion trap mass spectrometer equipped with an electrospray ionization source (AB SCIEX, Foster City, CA, United States) was introduced for the determination of the five mycotoxins. AFB_1_, B_2_, G_1_, and G_2_ and OTA were separated on an Agilent Poroshell 120 EC-C18 column (4.6 mm × 50 mm, 2.7 μm) run at 30°C. The mobile phase was consisted of (A) methanol (0.1% formic acid) and (B) 5 mmol/L ammonium acetate-water (0.1% formic acid) at a flow rate of 0.3 mL/min at an optimal gradient elution procedure: 60% B for 0–0.5 min, 5% B for 0.5–4.5 min, 60% B for 6.51–10 min. The conditions of mass spectrometer was as follows: nebulizer gas (GS1), 50 psi; auxiliary gas (GS2), 50 psi; curtain gas (CUR), 35 psi; capillary temperature, 550°C. To increase the ions specificity and minimize the background signal, the most intense ion-pair information for multiple reaction monitoring (MRM) detection was optimized as follows: *m/z* 313.0 → 285.1 and *m/z* 313.0 → 269.0 for AFB_1_, *m/z* 315.0 → 287.1 and *m/z* 315.0 → 259.1 for AFB_2_, *m/z* 329.0 → 243.1 and *m/z* 329.0 → 215.0 for AFG_1_, *m/z* 331.0 → 245.1 and *m/z* 331.0 → 217.0 for AFG_2_, and *m/z* 404.0 → 358.0 and *m/z* 404.0 → 239.1 for OTA.

### Determination of Four Bioactive Components

#### Sample Preparation

Approximately 250 mg of the non-infected and infected ginger samples collected at different intervals were weighed accurately and extracted with 10 mL of methanol by ultrasonication for 30 min in an ultrasonic cleaning bath at 100 W, 40 kHz. Thereafter, the extract was centrifuged at 10,000 rpm for 5 min, and the supernatant was filtered through a 0.22-μm syringe filter. Two microliters of the filtrate was injected into the ultra performance liquid chromatography (UPLC) system. All prepared solutions were stored at 4°C before analysis.

#### UPLC-PDA Conditions

The simultaneous determination of four bioactive components was performed by using an Acquity UPLC H-Class (Waters Corp., Milford, MA, United States) system equipped with photodiode array (PDA) detector. A Capcell Core C18 column (2.1 mm × 50 mm, 2.7 μm) at 30°C was used as the analytical column, and the mobile phases was consisted of (A) water and (B) acetonitrile at a flow rate of 0.25 mL/min at a linear gradient program: 0 min, 3% B; 10 min, 85% B; 15 min, 100% B; 18 min, 3% B; and finally, reconditioning to 3% B for 4 min. Measurement wavelength was set at 280 nm and each samples was measured in triplicate.

### Scanning Electron Microscopy Images

The scanning electron microscopy (SEM) images were provided for further morphological and microcosmic studies about ginger powders with the inoculation of fungi. Thirty-five percent formaldehyde solution was introduced for the fixing to the morphology of ginger and fungi for 3 h and washed with 0.1 M PBS (pH 7.2) for 20 min. The sample was dehydrated with a series concentration (30–100%) of ethanol for 0.5 h and kept for 1 h in ethanol. The samples were subjected to critical-point drying in CO_2_ and sprayed with gold in a metallizer. Then, the morphological characteristics of the samples were observed by using a field emission scanning electron microscope (JSM-6701F, JEOL, Japan) at a 10.0 kV acceleration voltage.

### Statistical Analysis

The experiments were performed independently for three times and the data has been expressed as mean ± standard deviation (SD), which were analyzed by analysis of variance (ANOVA). Statistical significance was defined as *P* < 0.05.

## Results and Discussion

### Changes of Inoculated Ginger Powders

**FIGURE 1:**
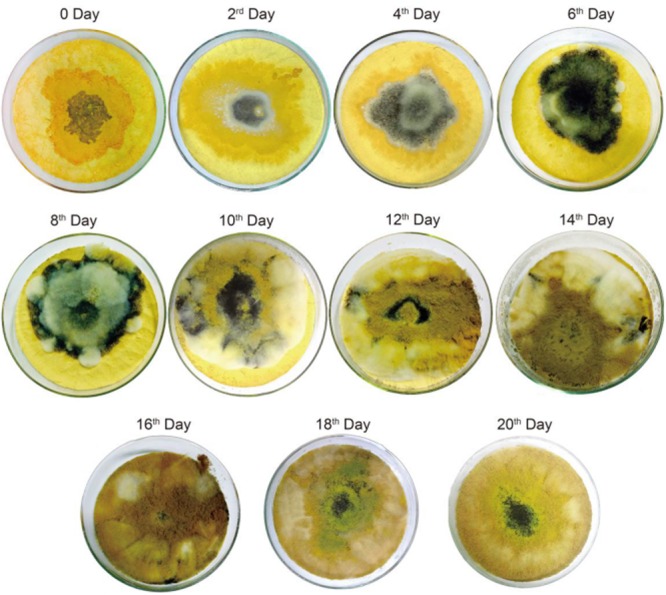
The surface variation of the infected ginger powders after fungal inoculation.

**FIGURE 2:**
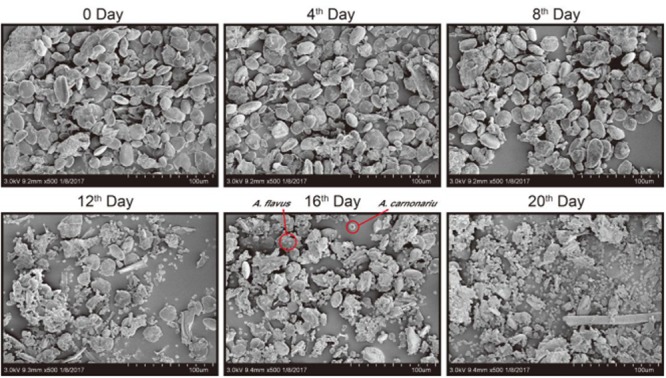
The SEM images of the infected ginger.

After being infected with the two fungi, the characteristic changes of the ginger powders were recorded from the pictures at a 2-day interval within 20 days in **Figure 1**. Yellowish white and black hyphae could be clearly seen with the increase of inoculation time. The ginger powder went to be moldy after being inoculated with a certain volume of *A. flavus* and *A. carbonarius* standard spore suspension for 2 days and become black in the center of inoculation circle 4 days later. With increasing the inoculation time, the moldy circle was filled out and the hyphae became darken. It was speculated that the nutrient substances in ginger were used by *A. flavus* and *A. carbonarius* for their growth. Furthermore, in the first 6-day duration, the *A. carbonarius* (the black mold) grew more vigorous than *A. flavus* (the white mold). However, in the next 12-day duration, the *A. carbonarius* were gradually covered up by *A. flavus*. So, it was inferred that when the two species of fungi existed simultaneously, *A. carbonarius* grew more quickly than *A. carbonarius* to produce more OTA than AFs in the ginger in the earlier stage of inoculation. The SEM images in **Figure 2** further exhibited the morphological changes of the infected ginger powders. It could be found that more fungi spores were adhered to the ginger particles. The spores of fungi were invisible after inoculation in the first 4 days, and a little number of spores could be observed at day 8 and then gave quick increase. Different sizes of spores of *A. flavus* (the smaller) and *A. carbonarius* (the larger) could be easily distinguished and the increase of *A. flavus* was more forceful than *A. carbonarius* in the middle-late stages which was consistent with the result of photo records in **Figure 1**.

### Determination of Mycotoxins in Ginger

#### UFLC-MS/MS Method Validation

The UFLC-MS/MS method was firstly validated in terms of linearity, stability, recovery, repeatability, and precision for accurate determination of the five mycotoxins.

A series of working solutions at different concentration levels (1, 5, 10, 20, 50, 100 ng/mL for AFB_1_, AFG_1_; 0.25, 1.25, 2.5, 5, 12.5, 25 ng/mL for AFB_2_, AFG_2_, and 10, 20, 50, 100, 500 ng/mL for OTA) were prepared by dissolving the toxins in methanol. Within their concentration ranges, the five analytes exhibited good linearity with correlation coefficients (*R*) higher than 0.9984, as well as low limits of detection and quantitation to show good sensitivity of the UFLC-MS/MS method. To evaluate the accuracy of the proposed method, recovery experiments were carried out and the average recoveries of the five analytes ranged from 89.52% to 112.71%. In addition, repeated experiments have shown the good stability (RSD < 3.92%), satisfactory precision (RSD < 8.80%) and reliable repeatability (RSD < 5.28%) of the established method.

Results have proved that the developed UFLC-MS/MS method was satisfactory for the determination of AFs and OTA in ginger after ultrasonic extraction and IAC clean-up.

#### Contents of Mycotoxins in Ginger

**FIGURE 3:**
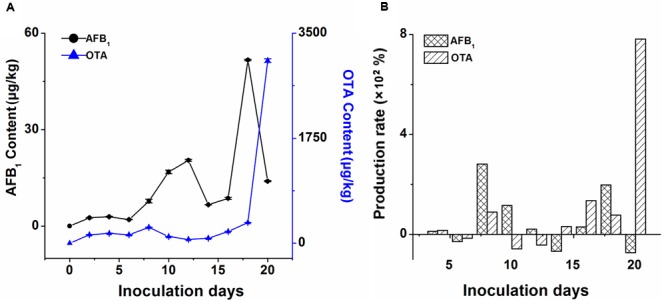
The plot of **(A)** mycotoxins variation and **(B)** production rate in ginger powders after fungal inoculation.

The contents of four AFs (AFB_1_, AFG_1_, AFB_2_, AFG_2_) and OTA in the non-infected ginger and infected ginger collected at a 2-day interval were determined by using the above validated UFLC-MS/MS method. AFs or OTA were not detected in the non-infected ginger samples, but, AFB1 and OTA were found at high contents in all the colleted samples infected by the fungi as shown in **Table 1**. The contents variation of AFB_1_ and OTA has been shown in **Figure 3A**. Low contents of AFB_1_ and OTA were detected at the early stage (he first 8 days) of inoculation of ginger. Then, their contents increased and OTA exhibited a drastic increasing trend 16 days later, which could be reflected from the SEM images in **Figures 1, 2**.

**Table 1:** Mycotoxin content in ginger powders after fungal inoculation (*n* = 3).

Days	AFB_1_ (μg/kg)	AFB_2_ (μg/kg)	AFG_1_ (μg/kg)	AFG_2_ (μg/kg)	OTA (μg/kg)
0	0.00 ± 0.00	0.00 ± 0.00	0.00 ± 0.00	0.00 ± 0.00	0.00 ± 0.00
2	2.59 ± 0.09	0.00 ± 0.00	0.00 ± 0.00	0.00 ± 0.00	142.46 ± 2.94
4	2.90 ± 0.13	0.00 ± 0.00	0.00 ± 0.00	0.00 ± 0.00	165.21 ± 4.15
6	2.05 ± 0.05	0.00 ± 0.00	0.00 ± 0.00	0.00 ± 0.00	139.98 ± 5.64
8	7.80 ± 0.58	0.00 ± 0.00	0.00 ± 0.00	0.00 ± 0.00	264.58 ± 4.22
10	16.87 ± 0.54	0.00 ± 0.00	0.00 ± 0.00	0.00 ± 0.00	111.57 ± 3.36
12	20.47 ± 0.31	0.00 ± 0.00	0.00 ± 0.00	0.00 ± 0.00	63.65 ± 1.85
14	6.63 ± 0.02	0.00 ± 0.00	0.00 ± 0.00	0.00 ± 0.00	83.30 ± 5.71
16	8.64 ± 0.31	0.00 ± 0.00	0.00 ± 0.00	0.00 ± 0.00	195.51 ± 0.94
18	51.69 ± 0.20	0.00 ± 0.00	0.00 ± 0.00	0.00 ± 0.00	345.58 ± 1.11
20	13.98 ± 0.18	0.00 ± 0.00	0.00 ± 0.00	0.00 ± 0.00	3045.37 ± 30.36

Another important parameter, the production rate, which could be used to reflect the accumulation of mycotoxins in the infect ginger, was calculated according to the following formula:

$$\begin{array}{c} \mathrm{Production}\;\mathrm{rate}\;(\%)\;=\;(C_{n}{\;}_{+}{\;}_{2}\;-\;C_{n})/C_{n}\times 100 \end{array}$$

where *C_n_* and *C_n_*_+2_ present the contents of mycotoxin in the infected ginger after inoculation of *A. flavus* and *A. carbonarius* for *n* and *n*+2 days, respectively. From **Figure 3B**, it could be found the increasing in production rate of OTA was higher than that of AFB_1_. During the first 4 days, the production rate were all low for AFB_1_ and OTA, and then increased with increasing the inoculation time within 20 days. The results could be reflected from the changes of pictures in **Figure 1**.

### Determination of Four Gingerol Components in Ginger

#### UPLC-PDA Method Validation

The UPLC-PDA method was also validated for linearity, stability, precision, repeatability, and recovery. A series of working solutions including 6-gingerol, 8-gingerol, 10-gingerol, and 6-shogaol at different concentration levels were prepared for the validation. Results showed that the four analytes within their concentration ranges expressed good linearity with correlation coefficients (*R*) higher than 0.9996 as well as low limits of detection and quantitation to show good sensitivity of the UFLC-PDA method. The average recoveries of them ranged from 97.76% to 99.74%. The relative standard deviations (RSDs) (%) for precision of intra- and inter-day were <3.39%, and for repeatability and stability validation were <3%. Therefore, the UPLC-PDA method was sensitive and accurate to be used to determine the four components of ginger.

#### Contents of Four Bioactive Components in Ginger

**FIGURE 4:**
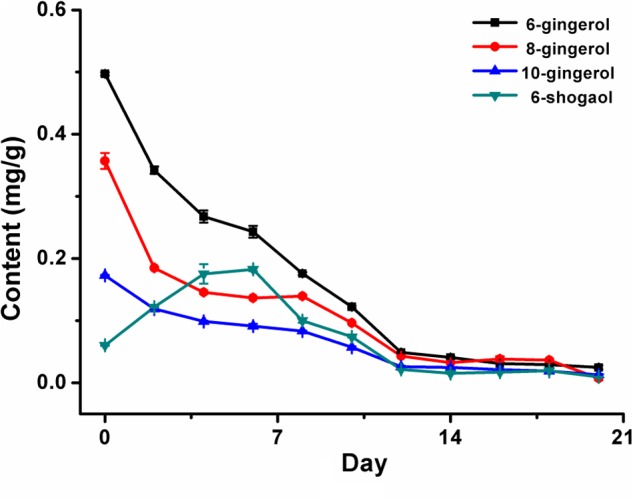
The plot of bioactive components variation in ginger powder after fungal inoculation for 20 days.

The developed UPLC-PDA technology was utilized for simultaneous determination of the four gingerol components in the infected ginger after inoculation with *A. flavus* and *A. carbonarius* and non-infected ginger in 20 days. The contents of 6-gingerol, 6-shogaol, 8-gingerol, and 10-gingerol in the non-infected ginger cultured in 20 days were basically same as that of infected ginger collected at 0 day. The results in **Table 2** and **Figure 4** showed that in the 20 days’ inoculation, the contents of 6-gingerol, 8-gingerol, and 10-gingerol decreased with increasing the inoculation time with the decreasing rate up to 95%, 98%, and 92.5%, respectively. While, the content of 6-shogaol increased in the first 6 days, and then decreased quickly.

**Table 2:** Bioactive components variation of ginger powders after fungal inoculation (*n* = 3).

Days	Contents (mg/g)			
	6-Gingerol	8-Gingerol	10-Gingerol	6-Shogaol
0	4.97 ± 0.67	3.57 ± 3.16	1.73 ± 0.26	0.60 ± 0.19
2	3.42 ± 1.49	1.85 ± 0.22	1.19 ± 0.28	1.22 ± 0.25
4	2.43 ± 2.36	1.37 ± 0.21	0.99 ± 0.34	1.83 ± 0.74
6	2.67 ± 2.49	1.46 ± 0.68	0.91 ± 0.94	1.51 ± 1.17
8	1.76 ± 0.61	1.39 ± 0.49	0.83 ± 0.13	1.00 ± 0.87
10	1.22 ± 0.49	0.96 ± 0.27	0.57 ± 0.42	0.74 ± 0.19
12	0.49 ± 0.25	0.43 ± 0.06	0.26 ± 0.36	0.21 ± 0.16
14	0.41 ± 0.58	0.38 ± 1.08	0.25 ± 0.12	0.15 ± 0.11
16	0.31 ± 0.11	0.36 ± 0.70	0.21 ± 0.26	0.17 ± 0.18
18	0.25 ± 0.81	0.32 ± 0.16	0.19 ± 0.37	0.19 ± 0.09
20	0.29 ± 0.57	0.07 ± 0.82	0.13 ± 0.02	0.10 ± 0.10

The results have indicated that fungal contamination will induce the decrease of the contents of some main bioactive components, which may further affect the quality of this TCM and the possible mechanism will be elucidated in the following study.

### Relationship of Mycotoxins Accumulation and Bioactive Components Variation in the Infected Ginger

From the above findings and the curves in **Figures 3, 4**, it could be concluded that a negative correlation was exhibited between the accumulation of AFs or OTA and the reduction in contents of four major bioactive components in the infected ginger by *A. flavus* and *A. carbonarius*. A certain linkage was existed between mycotoxins production and the contents reduction of active compounds. The reason might be that abundant nutrition in ginger was beneficial for fungal growth and the production of mycotoxins, which will further led to the decrease of the main bioactive components, to affect the inherent quality of ginger. The future work would focus on the production mechanism and metabolic process of mycotoxins for better understanding of the growth discipline and mycotoxins production and to take active measures to prevent mildew to warrant the quality and safety of ginger.

The relationship of mycotoxins accumulation and bioactive components variation in the infected ginger has not been studied before, but a similar conclusion was obtained that with the increasing of the contents of mycotoxins (AFs or OTA), the contents of major bioactive components decreased constantly in *G. uralensis* infected by *A. flavus* or *A. carbonarius* individually (Zhou et al., 2013, 2014). The differences were that waves can be seen in the changes of mycotoxins and the contents of 6-shogaol increased in the first 6 days in our study. The results implied that different matrixes would affect changes of contents of mycotoxins or bioactive components which might be related to nutritions in ginger. Another essential problem is that the competition or degradation may exist between the two kinds of moulds, which should be studied in the future.

### A New Component Detected

**FIGURE 5:**
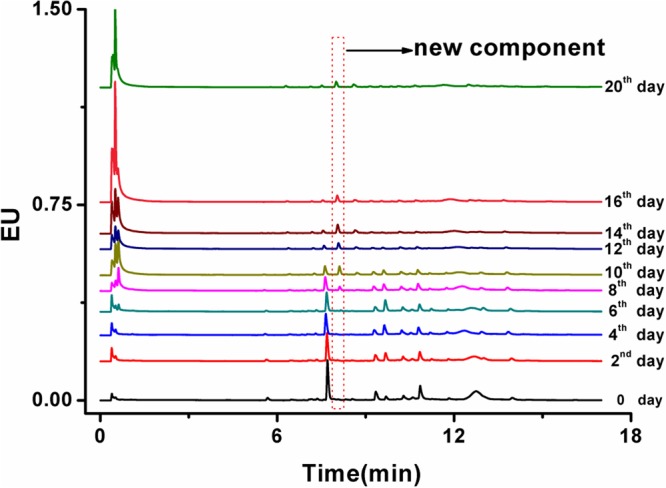
UPLC-PDA chromatogram of the infected ginger powders after fungal inoculation for 20 days.

**FIGURE 6:**
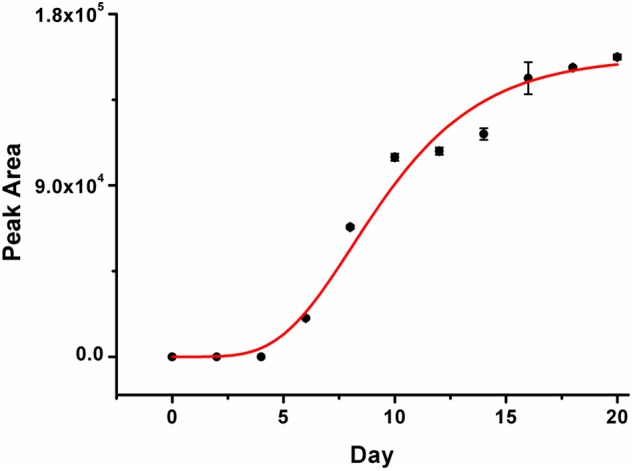
Peak area variation of one new compound in the infected ginger after fungal inoculation.

Some new phenomenon was observed in our study. From the chromatogram in **Figure 5**, one new chemical component was detected in ginger after inoculation for 6 days, which was determined temporarily as a new compound. The retention time of the new component was 8.10 min and the detected peak areas were listed in **Figure 6**. With increasing the inoculation time, the peak areas of the new component gave an increasing trend. The chemical structure and physicochemical property of it, as well as the relationship of the new component and fungi accumulation and other bioactive components would be explained in the future studies.

## Conclusion

In the present study, based on a novel trans-culturing mode and idea, the sterilized ginger was inoculated for 20 days with two toxigenic fungi to investigate the relationship of mycotoxins accumulation and bioactive components variation, as well as the effect of fungal contamination and the inherent quality of ginger. The results have shown that consecutive incubation of ginger with *A. flavus* and *A. carbonarius* have led to the accumulation of AFs (especially AFB_1_) and OTA, as well as the constant content reduction of four bioactive components. Negative correlation between the mycotoxins accumulation and bioactive components variation in ginger was found. In addition, one new by-product was detected in the infected ginger after inoculation for 6 days, which will be explained in the following study. All those observations were firstly reported. This study provided some useful references for the quality and safety assessment of TCMs and other matrices regarding its contamination by fungi and their metabolites such as mycotoxins.

## Author Contributions

YN, JL, and MY performed the research. WK and HW designed the research study. HW and GY performed the experiments. ZY, HW and GY analyzed the data. ZY wrote the paper.

## Conflict of Interest Statement

The authors declare that the research was conducted in the absence of any commercial or financial relationships that could be construed as a potential conflict of interest. The reviewer DP and handling Editor declared their shared affiliation, and the handling Editor states that the process nevertheless met the standards of a fair and objective review.
